# Comparative genome analysis identifies few traits unique to the *Escherichia coli* ST131 H30Rx clade and extensive mosaicism at the capsule locus

**DOI:** 10.1186/1471-2164-15-830

**Published:** 2014-09-30

**Authors:** Abdulaziz Alqasim, Fleming Scheutz, Zhiyong Zong, Alan McNally

**Affiliations:** Pathogen Research Group, Nottingham Trent University, Nottingham, NG11 8NS UK; Department of Microbiolgy and Infection, Statens Serum Institute, Copenhagen, Denmark; Department of Infection Control, West China Medical School, Chengdu, China

**Keywords:** *E. coli* ST131, Comparative genomics, Capsule, Recombination

## Abstract

**Background:**

*E.coli* ST131 is a globally disseminated clone of multi-drug resistant *E. coli* responsible for that vast majority of global extra-intestinal *E. coli* infections. Recent global genomic epidemiological studies have highlighted the highly clonal nature of this group of bacteria, however there appears to be inconsistency in some phenotypes associated with the clone, in particular capsule types as determined by K-antigen testing both biochemically and by PCR.

**Results:**

We performed improved quality assemblies on ten ST131 genomes previously sequenced by our group and compared them to a new reference genome sequence JJ1886 to identify the capsule loci across the drug-resistant clone H30Rx. Our data shows considerable genetic diversity within the capsule locus of H30Rx clone strains which is mirrored by classical K antigen testing. The varying capsule locus types appear to be randomly distributed across the H30Rx phylogeny suggesting multiple recombination events at this locus, but that this capsule heterogeneity has little to no effect on virulence associated phenotypes in vitro.

**Conclusions:**

Our data provides a framework for determining the capsular genetics of *E. coli* ST131 and further beyond to ExPEC strains, and highlights how capsular mosaicism may be an important strategy in becoming a successful globally disseminated human pathogen.

**Electronic supplementary material:**

The online version of this article (doi:10.1186/1471-2164-15-830) contains supplementary material, which is available to authorized users.

## Background

Extra-intestinal pathogenic *Escherichia coli* (ExPEC) infections are one of the leading causes of morbidity in the developed world and are particularly associated with infections of the urinary tract (UTI) and with bacteraemia. In recent years one particular clone of ExPEC has emerged to become a globally dominant cause of human infection, *E. coli* ST131 ([1] which is also associated with the emergence and spread of multiple-drug resistance in ExPEC infections via the sustained carriage of the CTX-M-15 extended spectrum beta-lactamase enzyme [2]. Recent work has focussed on elucidating the genomic epidemiology of this group of organisms since the report of the genetically homogeneous nature of clinically unrelated isolates in 2012 [3]. Two independent studies identified that all CTX-M-15 positive isolates belonged to a single expanded clone which emerged some time previous to 2000 [4, 5] and which is now referred to as the H30Rx clade of *E. coli* ST131 [4]. Both studies show this clade to be monomorphic containing a few dozen SNPs difference in data sets spanning geographical and temporal space.

The genetic architecture of the H30Rx clade was also examined [5], paying particular attention to virulence associated genes of ExPEC and to mobile genetic elements not found in non-ST131 ExPEC. In general these data suggested no ST131 specific virulence gene repertoire as such, though did highlight the seemingly unique nature of the second flagellar cluster Flag-2, which had been previously identified in *E. coli* ST131 genomes [3, 6]. Additionally the analysis also highlighted the role of intra-ST131 recombination in shaping the lineage [5] and identified a recombinant fragment common across ST131 within the capsule locus. Classical capsular typing of a collection of *E. coli* ST131 isolates, many of which were in the H30Rx clade, has shown high diversity in the biochemical profile of capsule antigens [7] which seems surprising given the monomorphic nature of the H30Rx clade. There were a total of 7 different K capsule types identified within the forty four ST131 isolates tested, which is in contrast to the vast majority of capsule typing which had been performed previously on *E. coli* ST131 using PCR based methods and which predominantly identified K2 type capsules via *kpsMII* primers [8, 9]. Indeed none of the strains biochemically tested were identified as K2 but rather as K100 despite testing K2 positive by PCR [7].

Given that the comparative genomics performed to date on *E. coli* ST131 have focussed on virulence associated genes, and the confusing data available to date on the diversity of the capsule locus, we sought to investigate loci uniquely associated with the H30Rx clade of *E. coli* ST131 using previously published genomes [3, 6, 10, 11]. We analysed a pangenome created from our ST131 genomes against reference non-ST131 ExPEC genomes to identify a small number of loci unique to ST131 dominated by lineage unique phages and the Flag-2 locus. Additionally we provide a genetic architecture for the diversity observed in the capsule locus of ST131, and show extensive genetic and biochemical diversity of the capsule region even within the H30Rx lineage of ST131. The random phylogenetic dispersal of these capsule loci suggests recombination occurs frequently at this region within ST131 and concurs with the previous suggestion that the capsule locus may be coming under strong selective pressure in the lifestyle of *E. coli* ST131 H30Rx [5].

## Results and Discussion

### Identification of genetic loci unique to the *E. coli*ST131 H30Rx clade

**Figure 1:**
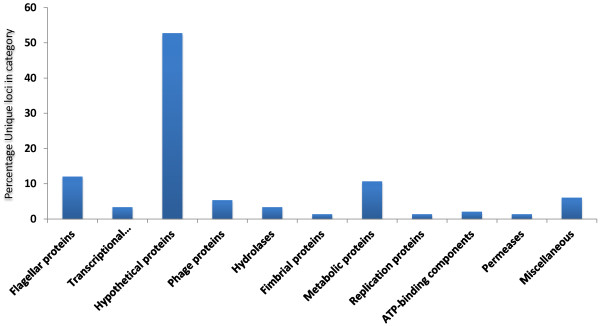
**Functional categories of genes unique to** ***E. coli*** **ST131 within the ExPEC pathotype.** Graph showing the distribution of functional categories for genes unique to the *E. coli* ST131 H30Rx compared to a collection of ExPEC reference genomes. Unique loci were identified by creating a pan genome of all genomes in Table 1, and then using the compare_bsr script in LS-BSR to identify loci unique to the ST131 H30Rx strains.

Given the focus on virulence associated genes in previous gene content studies, we aimed to determine loci unique to ST131 isolates without bias for functionality of the encoding loci. An ExPEC pan genome was constructed using the blast-score ratio method implemented in LS-BSR [12] containing twelve ST131 genomes and all available non-ST131 reference genome sequences (Table 1). Using the resulting pan-genome matrix we determined the genetic loci uniquely associated with the ST131 group versus the non-ST131 group using the compare_BSR python script implemented in the LS-BSR package. To define the ST131 group we excluded NA114 on the basis that previous work has suggested the methodology used to assemble the genome has resulted in regions missing from that genome that are present in all other H30Rx strains [5]. We also ran the analysis with SE15 as an ST131 but non-H30Rx strain to determine loci unique to the H30Rx clade to which SE15 does not belong [5, 13]. Our resulting data set identified a total of 150 loci unique to ST131 H30Rx strains in comparison to other ExPEC (Additional file 1), dominated by three phages common across the lineage and which have most probably been acquired by the common ancestral H30Rx progenitor and then maintained in the lineage. Hypothetical proteins dominate the functional category of genes (Figure 1) followed by flagellar associated genes and then a small number of metabolic loci. These metabolic loci correlate with data previously published by our group and add further weight to the assertion that ST131 H30Rx is not a metabolically distinct lineage [14]. The most striking locus with respect to potential biology is the confirmation that the Flag-2 accessory flagella locus is unique to ST131 H30Rx amongst ExPEC strains [5]. Again it is likely that this is ancestral to the H30Rx clade but its acquisition within the larger ST131 lineage is suggestive of a possible role in the formation and dissemination of the H30Rx clade and merits a fuller bacterial genetics investigation of its importance and role in the H30Rx clade. Indeed a fuller genetic investigation of all of the H30Rx loci identified as clade associated may be of merit. A saturated transposon mutant library has been constructed in an H30Rx strain and was utilised to determine the essential gene set for serum resistance [15]. Using such a library to test a wider set of environmental and infection conditions would undoubtedly elucidate if the H30Rx unique loci do indeed play a formative role in the success of the lineage.

**Table 1:** **List of strains and genomes used in this study**

Strain	ST	Pathotype	Accession number
*E. coli* UTI18	131	ExPEC	ERP001095
*E. coli* EC958	131	ExPEC	CAFL01000001
*E. coli* NA114	131	ExPEC	CP002797.1
*E. coli* UTI24	131	ExPEC	ERP001095
*E. coli* UTI32	131	ExPEC	ERP001095
*E. coli* UTI62	131	ExPEC	ERP001095
*E. coli* UTI188	131	ExPEC	ERP001095
*E. coli* UTI226	131	ExPEC	ERP001095
*E. coli* UTI306	131	ExPEC	ERP001095
*E. coli* UTI423	131	ExPEC	ERP001095
*E. coli* UTI587	131	ExPEC	ERP001095
*E. coli* UTI570	131	ExPEC	ERP001095
*E. coli* JIE168	131	ExPEC	ERP001095
*E. coli* JJ1886	131	ExPEC	CP006784.1
*E. coli* SE15	131	Human commensal	AP009378.1
*E. coli* LF82	135	AIEC	NC_011993.1
*E. coli* IHE3034	95	ST95 ExPEC	CP001969.1
*E. coli* UTI89	95	ST95 ExPEC	CP000243.1
*E. coli* S88	95	O45 ExPEC	CU928161.2
*E. coli* APEC01	95	APEC	CP000468.1
*E. coli* UM146	643	AIEC	CP002167.1
*E. coli* NRG857c	135	AIEC	CP001855.1
*E. coli* ED1a	452	O81	CU928162.2
ABU89372	73	Asymptomatic	CP001671
*E. coli* CFT073	73	ExPEC	AE014075.1
*E. coli* Di14	73	ExPEC	AE014075.1
*E. coli* Di12	73	ExPEC	CP002211.1

### Genetic architecture of capsule locus variation in the H30Rx clade

**Figure 2:**
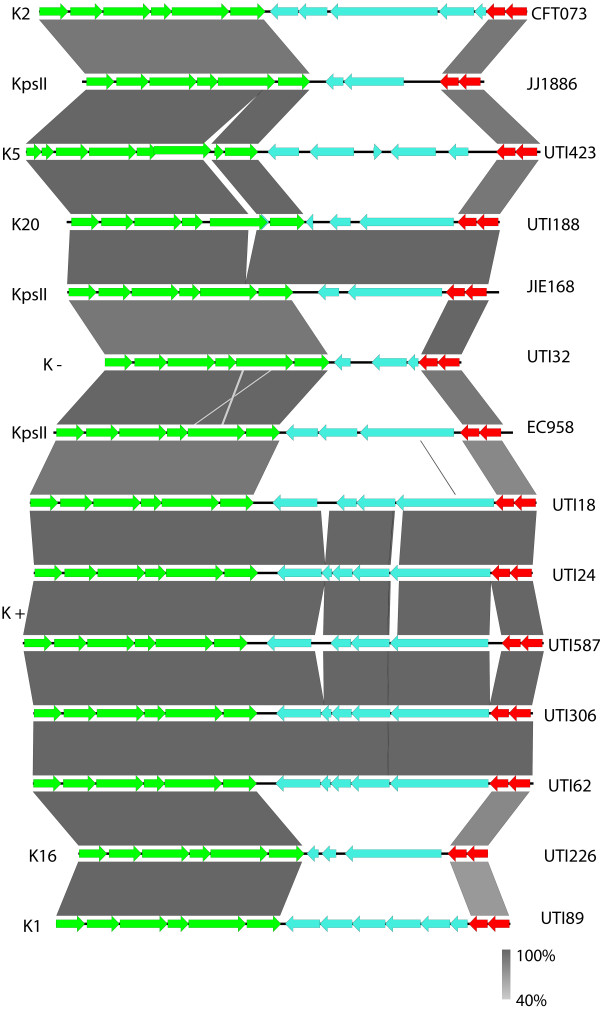
**Capsule locus genetics of a collection of** ***E. coli*** **ST131 H30Rx genomes.** Figure showing all of the capsule loci identified in available *E. coli* ST131 genomes. The CDS marked in green are the *kpsF-E-D-UC-S* cluster, and those in red the *kpsT-M* cluster, both of which are highly conserved across all *E. coli* capsules. The CDS marked in cyan are unique CDS to that capsule type. The grey shading indicates the level of identity between any given pair of CDS. The K antigen identified for each genetic capsule type is indicated in letters to the left, whilst the strain the capsule locus belongs to is indicated by letters to the right.

Given the reported variability of capsule loci [5] and capsular antigen type [7] in *E. coli* ST131 H30Rx clade strains, we investigated this locus in more detail. We selected the recently released JJ1886 genome [10] as our reference given it is the only ST131 genome sequenced and assembled to a standard of quality commensurate with being a high quality genome [16]. Using this reference we re-ordered the contigs of the ST131 genomes previously reported by our group [3] to ensure the genome architecture was as accurate as possible. We then identified the capsule loci of all of the ST131 genomes at our disposal and created separate embl files for each capsule locus of each strain which we then compared using EasyFig [17]. The comparison of the capsule loci (Figure 2) shows a high degree of diversity between the conserved *kpsS* and *kpsTM* regions, with no observable similarity between strains in the variable central genes. Blast analysis of each of the variable central genes in each genetic capsule type present returned no significant hits with any reference *E. coli* sequences. To ascertain how this genetic architecture reflected upon biochemical typing we determined the K antigen type of each of the genetic capsule types for strains which were in our possession by classical capsule typing (Figure 2), and also overlaid any available capsule type information on the other sequenced strains. Our data shows a correlation between the genetic capsular type and the biochemical typing data, and provides a framework for which to contextualise *E. coli* capsule types from genomic data. More importantly for this study our data clearly shows significant diversity within the capsule locus in *E. coli* ST131 H30Rx strains suggestive of frequent and targeted recombination in this region [5].

**Figure 3:**
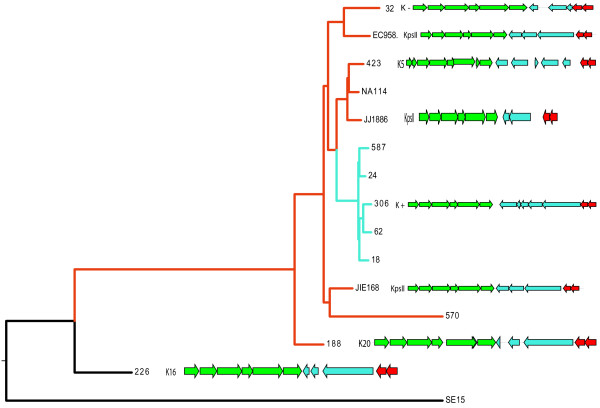
**Phylogenetic distribution of K-antigen types in** ***E. coli*** **ST131 H30Rx.** Core-genome phylogeny of the *E. coli* ST131 genomes analysed in this study, with the SE15 strain included as an outlier. The H30Rx strains are indicated by red colouration of tree branches. The Nottingham “outbreak” strains previously sequenced by our group are indicated by cyan colouration of the tree branches. The K-antigen type and accompanying capsule locus genetics are superimposed to the right of the tree.

To examine this in more detail we created a core genome phylogeny for *E. coli* ST131 using all the ST131 genome sequences in Table 1 and previously published methodology [13, 18] using SE15 as the root of the phylogeny given its phylogenetic position relative to H30Rx [5]. The resulting phylogenetic tree (Figure 3) confirms that all but one of the strains in our analysis, including those previously sequenced by our group prior to the discovery of H30Rx [3] do indeed belong the H30Rx clade. More importantly when the capsule loci genetics were superimposed on the phylogenetic tree it clearly demonstrates that the capsule loci are randomly distributed across the phylogeny. The only exceptions to this are the small cluster of strains containing UTI18 which have been previously shown by us to essentially be a single clone [3]. Such a random dispersal of the capsule loci across the phylogenetic tree can only be explained by extensive and targeted recombination events at this discreet location on the genome, suggesting there is some pressure acting on the capsule locus resulting in constant switching of capsule genes as the H30Rx clade evolves. Such extensive recombination has been well characterised in *Streptococcus pneumonia* where capsule locus switching has been shown to play a significant role in vaccine escape [19] and in the evolutionary dynamics of densely populated infection foci [20], however such dynamism in capsular recombination in *E. coli* is hitherto uncharacterised particularly in such a genetically monomorphic clade as ST131 H30Rx.

### Capsule diversity has no obvious effect on virulence associated phenotypes *in vitro*

Given the observation of extensive recombination at the capsule region of the H30Rx clade we sought to determine any obvious phenotypic effects. We compared the ability of our ST131 strains to form capsules at 25°C and 37°C on LB and CLED agar plates over a 14 day period. There was no association with the capsule loci present in the different H30Rx strains and levels of capsulation morphology on agar plates (Table 2). Classical 96 well plate biofilm formation assays also failed to show any significant pattern between different H30Rx capsular variants. We also conducted *in vitro* cell adhesion and invasion assays on T24 bladder epithelial cells using both the gentamicin protection assay to quantitate invasion, as well as confocal microscopy using strains carrying a medium copy number GFP^+^ containing plasmid [21]. As with our other virulence associated phenotypes there was no associated difference between different capsular variants of H30Rx. An identical pattern was also observed when the ability of the strains to survive inside cultured U937 macrophage like cell lines was assayed. Finally we determined the levels of serum resistance in our strains using methods previously employed in our lab [22, 23]. We found that the presence of different capsular variants had no effect on serum resistance and that all of our ST131 strains were totally resistant to serum in the 3 hours used for our assay (Table 2). The importance of serum resistance to *E. coli* ST131 has been documented and functionally characterised [15] with several glycosylation associated ORFs identified in as playing an essential role in serum resistance. Capsules have classically been considered as important factors in the ability of *E. coli* to survive human serum however our data suggests that that capsule type may be less important, and that the extensive capsular recombination demonstrated in the ST131 H30Rx clade has no effect on the ability of these pathogens to survive exposure to human serum. It may be that the capsule variability alters phenotypes important for in vivo environments, and there may be merit to future work investigating differences in infection dynamics between the capsule variants using appropriate surrogate infection models.

**Table 2:** **Results of capsule-associated phenotype tests for a selection of** ***E. coli*** **ST131 strains**

Strain	Capsule formation		Biofilm	Invasion of T24 cells	Serum resistance		Persistence in U937		Serotype
	37°C	25°C	37°C		T0	T3	T0	T24	
UTI18	+++	+++	+	++	7.67E + 06	1.17E + 08	2.44E + 05	8.56E + 04	O 25: K+: H-
UTI24	+++	+++	++	+	1.00E + 07	1.50E + 08	3.67E + 05	1.33E + 04	O 25: K+: H-
UTI32	++++	+++	+	+++	3.67E + 06	1.17E + 08	2.89E + 05	1.66E + 04	O 25: K-: H 4
UTI62	++	++	+	+	6.50E + 06	2.50E + 08	4.94E + 05	4.72E + 04	O 25: K+: H-
UTI188	++	++	+	++	5.67E + 06	2.67E + 08	3.61E + 05	1.28E + 05	O 25:K 20, K 23:H 4
UTI226	+++	++	+++++	++	4.17E + 06	1.00E + 08	3.11E + 05	1.56E + 04	O 25: K 16: H-
UTI306	+++	+++	+	+++	2.00E + 06	1.50E + 08	4.72E + 05	1.00E + 04	O 25: K+: H-
UTI423	+++	+++	++	++++	1.83E + 06	1.50E + 08	2.78E + 05	4.33E + 04	O 25: K 5: H 4
UTI570	++	++	+++++	++	1.00E + 07	1.17E + 08	4.22E + 05	2.17E + 04	NT
UTI587	++	++	++	++	4.33E + 06	4.83E + 08	3.72E + 05	8.33E + 04	O 25: K+: H-

Phenotype results are presented as an arbitrary score of: + low phenotypic expression, ++ medium phenotypic expression, +++ high phenotypic expression.

## Conclusions

*E. coli* ST131 is now the dominant causative agent of extra-intestinal infection by *E. coli* in the developed world, and is also heavily responsible for the increase in prevalence in multi-drug resistance in *E. coli* due to extended carriage of the CTX-M-15 ESBL gene [1]. Recent extensive genomic studies have led to a deep understanding of the phylogeography of this lineage of ExPEC [4, 5] and the discovery of a sub-clade of ST131 which is globally dominant and associated with the CTX-M-15 genotype which has been termed the H30Rx clade [4]. Despite these extensive studies the only efforts at comparative genomics of the ST131 lineage have focussed solely on virulence associated genes and large mobile genetic elements unique to the lineage [5]. Here we present an approach where we created an ExPEC pan-genome and then identified loci uniquely associated with the ST131 H30Rx clade. Our data is further suggestive that at a gene content level this clade is rather unremarkable in comparison to other ExPEC, as recently suggested for the clade at a metabolic level [14], with the secondary flagellar locus Flag-2 the stand-out region unique to ST131 within ExPEC. This region merits further detailed bacterial genetics analysis to uncover its true importance to the emergence and success of the H30Rx clade. Furthermore our analysis shows a surprising level of diversity within the capsule locus of the H30Rx clade with a phylogenetic distribution highly suggestive of frequent recombination at the locus. This recombination has no obvious detectable effect on virulence associated phenotypes *in vitro*. Given the level of diversity observed at the capsule locus it is tempting to speculate that there is significant selective pressure occurring at this site during the life cycle of the H30Rx clade, and that frequent recombination allows the clade to subvert that pressure. This has been documented to occur in other capsulated pathogens [20] and also ties in with previous data from our group showing that ST131 strains did not exhibit inter-species recombination across the *E. coli* species but that rather recombination events were focussed within the ST131 lineage [13]. Temporal studies of ST131 populations from patients and environmental reservoirs may allow us to determine if capsular switching does occur *in vivo* and if it is an important mechanism in the successful and prolonged dissemination of this important human pathogen.

## Methods

### Strains and genome data

A list of genomes used in our study is provided in Table 1, and of strains used in our study in Table 2. All strains have been previously characterised [3, 10, 13, 23] with the exception of strain JIE186, which is an Australian ST131 CTX-M-15 strain isolated in 2000, and has been submitted to the ENA under our existing ST131 study accession number ERP001095.

### Core and pan genome analysis

We created a pan genome for all ExPEC genomes in Table 1 using LS-BSR [12]. We then used the compare_BSR python script implemented in the LS-BSR package to identify loci unique to genomes belonging to the H30Rx clade, with the exception of NA114 which has been shown to have known H30Rx genes missing from its assembly [5]. The resulting 150 loci identified as H30Rx lineage unique were identified by performing BlastX searches against the genome of JJ1886 [10].

### Identification of capsule loci in ST131 genomes

FastQ sequencing data for all of the ST131 genomes produced by our group were re-assembled using Velvet and PAGIT [24] and using JJ1886 as a reference genome for contig re-ordering. This allowed us to re-order small contigs to the capsule region. The genomes were then annotated using Prokka [25] and the capsule regions written to new embl files using Artemis. The capsule encoding regions were visually compared using Easyfig [17] and variable genes were searched against the non-redundant database by BlastX search.

### Classical capsule typing

Serotyping was done according to the method of Ørskov and Ørskov. The K antigen was determined by countercurrent immunoelectrophoresis involving K-specific antisera, except for the K1 and K5 antigens, which were detected using K1- and K5-specific phages [7].

### Whole genome phylogeny

All ST131 genomes were aligned using Mugsy [26] and a core genome extracted as previously described [13, 18]. Maximum likelihood phylogeny was determined using RaxML [27] implementing the GTR-gamma model. The resulting phylogeny was visualised using Figtree.

### Phenotypic characterisation of strains

Biofilm formation was performed at 37°C in static cultures incubated for 5 days in both LB and BHI broth in a 96 well plate, with 5 wells per strain. Assays were performed on three independent occasions and values are representative values of measured levels of crystal violet retention as measured at A_600_. Capsule production was determined using a scoring system through testing the ability of each strain to form mucoid colonies in LB agar and in CLED agar plates using two incubation temperatures, 37°C and 25°C. Each strain was tested in triplicate. Ability to invade T24 bladder epithelial cells was performed as previously described [28], and also performed with strains carrying a GFP^+^ containing plasmid pMN402 [21] which were visualised using confocal microscopy. Serum resistance assays were performed as described previously [23], as were U937 macrophage cell line survival assays [21].

## Electronic supplementary material

Additional file 1: **Excel spreadsheet showing all of the loci identified as unique to our** ***E. coli*** **ST131 strains by our pan-genome analysis.** (XLSX 20 KB)

## References

[1] Rogers BA, Sidjabat HE, Paterson DL (2011). *Escherichia coli* O25b-ST131: a pandemic, multiresistant, community-associated strain. J Antimicrob Chemother.

[2] Johnson J, Tchesnokova V, Johnston B, Clabots C, Roberts P, Billig M, Riddell K, Rogers P, Qin X, Butler-Wu S, Price L, Aziz M, Nicolas-Chanoine M, Debroy C, Robicsek A, Hansen G, Urban C, Platell J, Trott D, Zhanel G, Weissman S, Cookson B, Fang F, Limaye A, Scholes D, Chattopadhyay S, Hooper D, Sokurenko E (2013). Abrupt emergence of a single dominant multidrug-resistant strain of *Escherichia coli*. J Infect Dis.

[3] Clark G, Paszkiewicz K, Hale J, Weston V, Constantinidou C, Penn CW, Achtman M, McNally A (2012). Genomic and molecular epidemiology analysis of clinical *Escherichia coli* ST131 isolates suggests circulation of a genetically monomorphic but phenotypically heterogeneous ExPEC clone. J Antimicrob Chemother.

[4] Price L, Johnson J, Aziz M, Clabots C, Johnston B, Tchesnokova V, Nordstrom L, Billig M, Chattopadhyay S, Stegger M, Andersen P, Pearson T, Riddell K, Rogers P, Scholes D, Kahl B, Keim P, Sokurenko E (2013). The epidemic of extended-spectrum-β-lactamase-producing *Escherichia coli* ST131 is driven by a single highly pathogenic subclone, H30-Rx. MBio.

[5] Petty N, Ben Zakour N, Stanton-Cook M, Skippington E, Totsika M, Forde B, Phan M, Gomes Moriel D, Peters K, Davies M, Rogers B, Dougan G, Rodriguez-Baño J, Pascual A, Pitout J, Upton M, Paterson D, Walsh T, Schembri M, Beatson S (2014). Global dissemination of a multidrug resistant *Escherichia coli* clone. Proc Natl Acad Sci U S A.

[6] Totsika M, Beatson SA, Sarkar S, Phan MD, Petty NK, Bachmann N, Szubert M, Sidjabat HE, Paterson DL, Upton M, Schembri MA (2011). Insights into a multidrug resistant *Escherichia coli* pathogen of the globally disseminated ST131 lineage: genome analysis and virulence mechanisms. PLoS One.

[7] Olesen B, Hansen D, Nilsson F, Frimodt-Møller J, Leihof R, Struve C, Scheutz F, Johnston B, Krogfelt K, Johnson J (2013). Prevalence and characteristics of the epidemic multiresistant *Escherichia coli* ST131 clonal group among extended-spectrum beta-lactamase-producing E. coli isolates in Copenhagen, Denmark. J Clin Microbiol.

[8] Johnson J, O’Bryan T (2004). Detection of the *Escherichia coli* group 2 polysaccharide capsule synthesis Gene *kpsM* by a rapid and specific PCR based assay. J Clin Microbiol.

[9] Croxall G, Hale J, Weston V, Manning G, Cheetham P, Achtman M, McNally A (2011). Molecular epidemiology of extraintestinal pathogenic *Escherichia coli* isolates from a regional cohort of elderly patients highlights the prevalence of ST131 strains with increased antimicrobial resistance in both community and hospital care settings. J Antimicrob Chemother.

[10] Andersen P, Stegger M, Aziz M, Contente-Cuomo T, Gibbons H, Keim P, Sokurenko E, Johnson J, Price L (2013). Complete genome sequence of the epidemic and highly virulent CTX-M-15-producing H30-Rx Subclone of *Escherichia coli* ST131. Genome Announc.

[11] Avasthi TS, Kumar N, Baddam R, Hussain A, Nandanwar N, Jadhav S, Ahmed N (2011). Genome of multidrug-resistant Uropathogenic *Escherichia coli* Strain NA114 from India. J Bacteriol.

[12] Sahl J, Caporaso J, Rasko D, Keim P (2014). The large-scale blast score ratio (LS-BSR) pipeline: a method to rapidly compare genetic content between bacterial genomes. PeerJ.

[13] McNally A, Cheng L, Harris SR, Corander J (2013). The evolutionary path to extra intestinal pathogenic, drug resistant *Escherichia coli* is marked by drastic reduction in detectable recombination within the core genome. Genome Biol Evol.

[14] Alqasim A, Emes R, Clark G, Newcombe J, La Ragione R, McNally A (2014). Phenotypic microarrays suggest Escherichia coli ST131 is not a metabolically distinct lineage of extra-intestinal pathogenic E. coli. PLoS One.

[15] Phan M, Peters K, Sarkar S, Lukowski S, Allsopp L, Gomes Moriel D, Achard M, Totsika M, Marshall V, Upton M, Beatson S, Schembri M (2013). The serum resistome of a globally disseminated multidrug resistant uropathogenic Escherichia coli clone. PLoS Genet.

[16] Chain PS, Grafham DV, Fulton RS, Fitzgerald MG, Hostetler J, Muzny D, Ali J, Birren B, Bruce DC, Buhay C, Cole JR, Ding Y, Dugan S, Field D, Garrity GM, Gibbs R, Graves T, Han CS, Harrison SH, Highlander S, Hugenholtz P, Khouri HM, Kodira CD, Kolker E, Kyrpides NC, Lang D, Lapidus A, Malfatti SA, Markowitz V, Metha T (2009). Genomics: genome project standards in a new era of sequencing. Science.

[17] Sullivan M, Petty N, Beatson S (2011). Easyfig: a genome comparison visualizer. Bioinformatics.

[18] Sahl JW, Johnson JK, Harris AD, Phillippy AM, Hsiao WW, Thom KA, Rasko DA: **Genomic comparison of multi-drug resistant invasive and colonizing*****Acinetobacter baumannii*****isolated from diverse human body sites reveals genomic plasticity.** *BMC Genomics* 2011.,**12**(291)**:** doi:10.1186/1471–2164–12–291 10.1186/1471-2164-12-291 PMC3126785 21639920

[19] Croucher N, Finkelstein J, Pelton S, Mitchell P, Lee G, Parkhill J, Bentley S, Hanage W, Lipsitch M (2013). Population genomics of post-vaccine changes in pneumococcal epidemiology. Nat Genet.

[20] Chewapreecha C, Harris S, Croucher N, Turner C, Marttinen P, Cheng L, Pessia A, Aanensen D, Mather A, Page A, Salter S, Harris D, Nosten F, Goldblatt D, Corander J, Parkhill J, Turner P, Bentley S (2014). Dense genomic sampling identifies highways of pneumococcal recombination. Nat Genet.

[21] McNally A, Dalton T, Ragione RML, Stapleton K, Manning G, Newell DG (2006). *Yersinia enterocolitica* isolates of differing biotypes from humans and animals are adherent, invasive and persist in macrophages, but differ in cytokine secretion profiles in vitro. J Med Microbiol.

[22] Alhashash F, Weston V, Diggle M, McNally A (2013). Multidrug-Resistant *Escherichia coli* Bacteremia. Emerg Infect Dis.

[23] McNally A, Alhashash F, Collins M, Alqasim A, Paszckiewicz K, Weston V, Diggle M (2013). Genomic analysis of Extra-intestinal pathogenic *Escherichia coli* urosepsis. Clin Microbiol Infect.

[24] Swain MT, Tsai IJ, Assefa SA, Newbold C, Berriman M, Otto TD (2012). A post-assembly genome-improvement toolkit (PAGIT) to obtain annotated genomes from contigs. Nat Protoc.

[25] Seemann T (2014). Prokka: rapid prokaryotic genome annotation. Bioinformatics.

[26] Angiuoli SVSS (2011). Mugsy: fast multiple alignment of closely related whole genomes. Bioinformatics.

[27] Stamatakis A, Ludwig T, Meier H (2005). RAxML-III: a fast program for maximum likelihood-based inference of large phylogenetic trees. Bioinformatics.

[28] Croxall G, Weston V, Joseph S, Manning G, Cheetham P, McNally A (2011). Increased Human Pathogenic Potential of *Escherichia coli* from Polymicrobial Urinary Tract Infections in Comparison to Isolates from Monomicrobial Culture Samples. J Med Microbiol.

